# Epilepsy and Neuromodulation—Randomized Controlled Trials

**DOI:** 10.3390/brainsci8040069

**Published:** 2018-04-18

**Authors:** Churl-Su Kwon, Valeria Ripa, Omar Al-Awar, Fedor Panov, Saadi Ghatan, Nathalie Jetté

**Affiliations:** 1 Department of Neurology, Icahn School of Medicine at Mount Sinai, New York, NY 10029, USA; nathalie.jette@mssm.edu; 2 Department of Population Health Science and Policy, Icahn School of Medicine at Mount Sinai, New York, NY 10029, USA; 3 Department of Neurosurgery, Icahn School of Medicine at Mount Sinai, New York, NY 10029, USA; fedor.panov@mountsinai.org (F.P.); Saadi.Ghatan@mountsinai.org (S.G.); 4 St. George’s Medical School, St. George, Grenada; valeriaripa87@gmail.com; 5 Department of Neurosurgery, Oxford University, John Radcliffe Hospital, Oxford OX3 9DU, UK; awar_omar@hotmail.com

**Keywords:** epilepsy, neuromodulation, randomized clinical trials (RCT), deep brain stimulation (DBS), transcranial direct current stimulation (tDCS), vagal nerve stimulation (VNS), external trigeminal nerve stimulation (eTNS), repetitive transcranial magnetic stimulation (rTMS), responsive neurostimulation (RNS)

## Abstract

Neuromodulation is a treatment strategy that is increasingly being utilized in those suffering from drug-resistant epilepsy who are not appropriate for resective surgery. The number of double-blinded RCTs demonstrating the efficacy of neurostimulation in persons with epilepsy is increasing. Although reductions in seizure frequency is common in these trials, obtaining seizure freedom is rare. Invasive neuromodulation procedures (DBS, VNS, and RNS) have been approved as therapeutic measures. However, further investigations are necessary to delineate effective targeting, minimize side effects that are related to chronic implantation and to improve the cost effectiveness of these devices. The RCTs of non-invasive modes of neuromodulation whilst showing much promise (tDCS, eTNS, rTMS), require larger powered studies as well as studies that focus at better targeting techniques. We provide a review of double-blinded randomized clinical trials that have been conducted for neuromodulation in epilepsy.

## 1. History of Neuromodulation

There has been a long experimental history of cortical and deep brain neuromodulation in epilepsy. Only in the last 20 years however, through improved knowledge of brain networks, accurate stereotactic neurosurgery and robust trial design has this been translated into accepted clinical use. The dawn of human stereotactic deep brain stimulation (DBS) can be seen in medial thalamotomy psychosurgery by Spiegel and Wycis in 1947 in an attempt to decrease the brutal but commonly performed frontal lobotomies [1]. Gildenberg, a fellow at the time to Spiegel and Wycis, stated that intraoperative brain stimulation was used as a means of investigating the target prior to lesioning. Hence, from the origins of DBS, electrical stimulation was adopted as a physiological tool to evaluate deep brain structures [2]. Epilepsy too became an interest amongst early DBS pioneers, and initial studies looked to target epileptic foci with implantation of chronic stereotactic deep brain electrodes for interrogation and intermittent stimulation [3]. In 1947, the first human stereotactic apparatus was designed and used by Talairach in order to record and stimulate temporal structures in patients with epilepsy [4]. Cooper pioneered therapeutic chronic stimulation for epilepsy in the early 1970s with a focus on the cerebellum due to the existing evidence of the inhibitory effects of this structure. 

An additional early target for epilepsy was the anterior nucleus of the thalamus (ANT). In fact, as early as 1979, Cooper implanted chronic ANT DBS electrodes in patients with drug-resistant focal impaired awareness seizures [5]. This very same structure has resurfaced recently in a double-blinded RCT showing clinical benefit in some adults with drug-resistant focal seizures and focal to bilateral tonic clonic seizures [6]. Velasco and colleagues in 1987 targeted the centromedian thalamic nuclei for treatment of generalized or multifocal uncontrollable seizures in five patients, where clinical seizures were significantly reduced as were EEG interictal spikes and slow waves [7]. The author performed further studies addressing the role of the centromedian thalamic nuclei in epilepsy pathogenesis and examining the long term effect of chronic electrical stimulation [8,9,10]. 

The technique of chronic stimulation of subcortical structures was proposed soon after the introduction of human stereotactic surgery in 1947 [11]. What was first used as a tool to study and treat neuropsychiatric disorders led to applications in pain management, then epilepsy, and finally movement disorders. The historical evidence presented challenges the notion of more recent published studies declaring DBS “a new approach” in epilepsy treatment [11]. 

## 2. Mechanism

Mechanisms of action of neuromodulation for epilepsy control are poorly understood and acknowledged as multifaceted and multifarious. Stimulation parameters used in clinical trials have been commonly experimental in nature, often derived from subjective configurations investigated with each anatomic structure in question. A chasm is still evident between insufficient animal data and limited clinical models. A need exists for more studies looking into the optimal stimulation parameters for the clinical management of seizures. 

Early animal models by Ranck investigated the amounts of current necessary to stimulate various myelinated and unmyelinated neural structures within the central nervous system, showing that electrical fields have a variance in effect on different neuronal structures [12]. A proposed mechanism of action based on studies performed by Velasco and colleagues suggested that high frequency stimulation of kindled neuronal structures increases after-discharge thresholds with subsequent seizure reduction [13]. Additionally, it was noted that high frequency stimulation reduced regional cerebral blood supply in the stimulated area and in the centromedian thalamic nucleus, causing suppression of thalamic and cortical spike-wave and synchronous firing. Parahippocampal cortex high frequency stimulation was seen to enhance the GABAergic benzodiazepine receptor numbers in the operated field [13]. Interestingly, low-frequency stimulation had the potential to trigger or exacerbate epilepsy in some susceptible areas, but had an inhibitory effect in others [13,14].

Some authors speculate that direct electrical current may have inhibitory effects on neurons that participate in initiation, propagation, and protraction of epileptic activity in certain anatomical regions [15]. Such inhibitory effects may be due to high extracellular potassium accumulation post high frequency stimulation, causing depolarization of neurons and tonic inactivation of sodium channels, further prohibiting initiation of action potential and consequently seizure activation and propagation. Small elevations of potassium are capable of lowering the seizure threshold, but large increases cause an opposite reaction and thus inhibit pathological bursting [15]. 

Other important studies have shown that the mechanism of seizure inhibition may be more complex and possibly involves alteration of gene expression and protein synthesis [14]. The antiepileptogenic effects of low-frequency stimulation have been associated with the attenuation of adenosine receptor gene expression via inhibition of the dentate gyrus and chronic vagal nerve stimulation is seen to alter various amino acids and neurotransmitters in the brain, with decreases in aspartate (excitatory amino acid), increases in GABA (inhibitory), and increases in ethanolamine, a membrane lipid precursor [16]. 

Further mechanisms of action regarding each modulation intervention are described in their respective sections.

## 3. Modes of Treatment and Anatomical Targets for Stimulation in Epilepsy

Epilepsy affects 1% of the population worldwide, and approximately 30% of patients are drug-resistant [17,18]. Other than the few candidates for resective surgery, most will have persistent often disabling seizures for the rest of their lives [19]. Neuromodulation is an alternative treatment strategy for patient with drug-resistant epilepsy. It is most often used in those for whom resective surgery is not feasible (i.e., very extensive network, multifocal epilepsy, or epileptogenic zone in eloquent cortex). 

There is much heterogeneity in the modes of electrical stimulation for the treatment of epilepsy, with a variety of anatomical structures, stimulation parameters, and outcome measures. Further discussion will cover structures and neuromodulation modes of treatment for epilepsy with a focus on published double-blinded RCTs (Table 1).

### 3.1. Deep Brain Stimulation (DBS)

Cerebellum: The earliest target of deep brain stimulation was the cerebellum. Therapeutic chronic stimulation became a treatment modality for epilepsy in the early 1970s pioneered by Cooper, and targeted due to the existing evidence of the inhibitory effects of this structure [20]. In his study, 10 of 15 patients with drug-resistant epilepsy had significant reduction or complete seizure inhibition during three years of chronic anterior lobe cerebellar stimulation [20]. According to Rosenow, in another group, Cooper targeted the anteromedial cerebellar surface for electrode placement and was able to achieve >50% reduction in seizure frequency in 18 of 32 patients [5]. Following these results, Van Buren presented a double-blinded cerebellar stimulation study of five patients with drug-resistant seizures that showed no significant difference in outcome [21]. Due to the resultant uncertainty surrounding the long-term outcomes of cerebellar stimulation, Wright et al. performed a double-blinded trial of chronic cerebellar stimulation in 12 patients with severe epilepsy [22]. Two 8-button pads were positioned on the upper surface of the cerebellum providing a mean peak current of 5–7 mA and frequency of 10 cathodal pulses per second of alternating polarity. Two of the patients had more bespoke parameters based on their responses. The patients received three modes of stimulation with randomly allocated two month phases of (1) continuous stimulation, (2) intermittent contingent stimulation, and (3) no stimulation. There was no significant reduction in seizure frequency in any of the groups within this trial [22]. After these results, cerebellar stimulation fell out of favor, until Velasco et al. re-evaluated the controversial topic with the aid of improved technology from radiofrequency-linked pulse generators to a fully implantable programmable battery-operated pulse generator [23]. Five drug-resistant epilepsy patients were involved in the study with insertion of two four-contact plates onto the supero-medial cerebellar surface. Fixed pulse width of 0.45 ms with current at 3.8 mA producing a charge density of 2.0 µC/cm^2^/phase was utilized. Pulse frequency was 10 pulses per second as used in the prior trials. The patients served as their own controls and in the initial three months double-blinded stage, a 33% reduction in seizures was reported in those with the stimulation initially on. All five patients in the unblinded stimulation period at six months had a mean seizure reduction rate of 41%, with significant reductions in tonic-clonic seizures and tonic seizures. Adverse events were all infectious in nature [23]. 

The differing target areas of stimulation and seizure patterns may be a reason for the variances seen in these studies. However, positive results seen in certain trials show that the cerebellum remains a potential target for neuromodulation in epilepsy. 

Centromedian nucleus of the thalamus (CMT): As part of the cortico–striato–thalamic pathway, the CMT has extensive projections to the cortex and has been observed to be involved in cortical excitation and seizure propagation [24,25]. Several pioneering studies have shown seizure reduction and decreased frequency in EEG spiking for generalized epilepsy [7,26,27,28,29]; other case reports have also proven its clinical effectiveness in refractory status epilepticus [30,31].

Two double-blinded RCTs have been performed on this structure. Fisher et al. performed in six patients a cross-over on/off stimulation protocol in three month blocks with a three month washout period. A 30% reduction in tonic-clonic seizure frequency with stimulation vs. 8% reduction in the sham period was noted using stimulator parameters of 90 µs pulses at 65 pulses/s, 1 min of each 5 min, for 2 h/day. To maintain effective blinding, the stimulation amplitude was set to 50% of sensory threshold. No statistically significant improvement was seen in the double-blinded phase of study. However, thresholds were increased to 90% in the open phase follow-up of the investigation, where three of the six patients saw a >50% reduction in generalized seizure frequency. Velasco et al. performed in 13 patients a double-blinded 6 month cross-over protocol with 3 month period of no stimulation (between 6–12 months after implantation; when stimulating-alternating right and left paradigm using parameters of 60 Hz, 4–6 V. 1 min of each 5 min, for 24 h/day) [27]. No significance was seen in mean seizure frequency reduction. Long-term open-label follow-up however showed a mean seizure reduction of 81.6% in patients with Lennox-Gastaut syndrome compared to 57.3% in patients with focal epilepsy [27].

Anterior nuclei of the thalamus (ANT): As part of the limbic system, the ANT is connected to the hippocampus and receives projections from the mammillary bodies via the mamillothalamic tract and fornix, while itself projecting to the cingulate gyrus, orbito-frontal, and mesial prefrontal cortices [32]. Neuronal activity of the thalamic nuclei includes two major types of discharge: tonic and burst-firing. Additionally, theta activity is noted in some neurons and thought to play an important role in synaptic plasticity of the hippocampal circuit. Based on animal studies, and its central location with abundant connectivity, the ANT became a common and attractive target for DBS for the treatment of drug-resistant epilepsy. ANT DBS is an approved target for therapy in Europe in treating focal epilepsy for adults, whereas it is still awaiting approval from the United States Food and Drug Administration (FDA). 

Cooper and Upton first published subjects who underwent ANT DBS for drug-resistant focal impaired awareness seizures in 1985. Five of six patients had a reduction of more than 60% in seizure frequency with stimulation at 3.5 V and 60–70 Hz [5]. Following this study, various case series reported a mean seizure reduction of >50% after ANT DBS [33,34,35,36,37,38,39]. These results subsequently led to a large double-blinded RCT for the Stimulation of the Anterior Nucleus of Thalamus for Epilepsy (SANTE) in patients with drug-resistant focal epilepsy, of which more than half of the study subjects had prior epilepsy or VNS surgery. Data from 110 patients with focal seizures focal to bilateral tonic-clonic seizures were collected [6]. Bilateral leads were implanted and after a one month post-operative baseline period, patients were randomized to receive three months of stimulation with duration 90 µs, frequency 145 Hz, at 5 V (on for one minute then off for five minutes) vs. no stimulation. After the double-blinded period, all participants received nine months of stimulation. Long-term follow-up showed a median seizure reduction from baseline at one year of 41% to 69% at five years. In the five years of follow-up, 16% of patients were seizure-free. Quality of life significantly improved from baseline at year one and five. Adverse effects included stimulation-related paresthesia (22.7%), implant site pain (20.9%), and infection (12.7%). Other open-label studies reported a reduction in seizure frequency >50%, however insertion effects could not be ruled out due to the nature of study design [36,38,40]. 

Hippocampus (HCP): Focal epilepsy involving the temporal lobe is well known to be the most resistant to pharmacological treatment. Even though surgical resection of the epileptic focus achieves more than 70% success rate in appropriately selected patients, this treatment option is not feasible for patients with bilateral disease or in those where resection would necessitate removal of the critical amygdalo-hippocampal complex responsible for verbal memory [18,41,42]. In such patients, the hippocampus serves as an appealing target for neuromodulation, and HCP sclerosis is known to be the most responsive to surgical treatment with favorable outcome [43,44]. 

Experiments on low-frequency amygdalo-hippocampal stimulation were initiated in the 1990s and showed profound effect on seizure development, expression, and thresholds, where it has been proposed that chronic low-frequency stimulation inhibits kindling [45,46]. Certainly, in long-term animal models, chronic low-frequency stimulation is associated with suppressed inhibitory effects over time and thus increased seizure thresholds [47,48]. Alternatively, a proposed mechanism for the effectiveness of high frequency hippocampal stimulation for epilepsy includes increased after-discharge thresholds and latencies that shorten the duration of the after discharges, which then reduce excitability as well as inducing hypoperfusion of the amygdalo-hippocampal complex [49,50,51]. 

Five RCTs evaluated hippocampal DBS [52,53,54,55,56]. The first by Tellez-Zenteno et al. performed a double-blind cross over RCT in four patients with refractory mesial temporal lobe epilepsy (MTLE) [52]. A median seizure reduction of 15% (not significant) was observed comparing stimulation vs. no stimulation. Seizure severity and symptomatology did not change [52]. Velasco et al. proceeded to a one month blinded trial in nine MTLE patients and reported a median seizure frequency reduction of 40% in the stimulation group vs. 0% in the no stimulation group (graphical presentation) [53]. However, neither numerical values were given nor were the statistical significance of the results stated [53]. McLachlan et al. also performed a cross-over RCT in two MTLE patients with mean reduction in seizures of 33% in those with stimulation [54]. Although, these three studies showed decreases in seizure frequency, sample sizes were too small and there was variability between the studies in placebo effect [52,53,54]. Thus, a larger trial was designed by Wiebe et al. who looked to examined whether hippocampal DBS was more safe and effective than simply implanting an electrode without stimulation [55]. Despite being a multicenter trial, only six out of a target sample of 57 were recruited leading to a halt in the RCT. None of the outcomes were statistically significant, likely due largely to the small sample size [55]. Cukiert et al. recently published a double-blinded RCT of hippocampal DBS in patients with drug-resistant temporal lobe epilepsy. Sixteen patients were randomized 1:1 to either stimulation or no stimulation. All patients received bipolar continuous stimulation with duration of 300 µs, frequency at 130 Hz and weekly 0.4 V stimulus intensity increments to a maximum of 2 V. A significant reduction in seizure frequency was observed in the stimulation group at full generator activation of 2 V. Half of the active group became seizure free. Seven of the eight participants had at least 50% reduction in seizure frequency. Local skin erosions were the main side-effects noted in this study [56]. Other open-label studies quoted seizure freedom rates of 15–45% [53,57,58,59]. 

The mechanisms involved in the reduction of seizures, using optimal stimulating parameters and targets are still unclear for hippocampal DBS. Further investigations into these variables are critical in delineating its potential benefit. 

Nucleus Accumbens (NAc): This structure has an important role in both the anatomical and functional connectivity between frontal and temporal lobes [60]. In animal models, the NAc is seen to be involved in the propagation of epileptiform activity [61,62]. In the only RCT of this structure performed by Kowski et al. 4 patients underwent a cross-over protocol with bilateral DBS implantation of the NAc and ANT. One month post-surgery the patients were randomized to receive NAc stimulation or no stimulation (125 Hz, 5 V, 90 µs, 1 min stimulation/5 min off). The treatment protocol lasted three months and after a one month washout period the patients switched to the other protocol. The ANT was continuously switched on in all patients. Three out of the four patients experienced >50% reduction in frequency of disabling seizures with no further improvement with additional ANT stimulation [63]. These results will need to be further interrogated by higher powered studies in the future.

New DBS targets are continuously being identified and characterized for patients with difficult to treat epilepsy. The subthalamic nucleus (STN) has an important role in motor control and motor-related seizures and is thought to desynchronize motor pathways [64]. Several small case series exist of STN DBS inserted for cases of motor-related seizures with favorable outcome [65,66,67]. Following these small successes, a double-blinded RCT (STIMEP trial) was put in place. However, this was terminated due to insufficient enrollment. The caudate nucleus has also been seen as a target due to its involvement in the cortico–striato–thalamic pathway and rationale of inducing cortical hyperpolarization via neuromodulation [68,69]. Chkhenkeli et al. exhibited in a subset of their large cohort of patients that with low frequency 4–8 Hz stimulation, cortical and hippocampal interictal spiking and epileptiform activity decreased. However, due to the heterogeneity of the population, varying stimulation protocols, uncontrolled observations, and short follow-up it is difficult to properly interpret these studies.

### 3.2. Transcranial Direct Current Stimulation (tDCS)

tDCS is an emerging noninvasive stimulation technique that modulates cortical activity [70]. It utilizes weak direct current to modulate neuronal membrane potentials and hence cortical activity. The continuous stimulation in turn displaces polar-sensitive molecules, neurotransmitters, and receptors in brain tissue, triggering a polarity shift in membrane potentials [70,71]. Negative cathodal stimulation is proposed to cause cortical inhibition and diminish epileptiform discharges. The only double-blinded RCT compared cathodal tDCS at 2 mA for 30 min over the epileptic foci in three settings: three consecutive days, five consecutive days, and placebo stimulation [72]. In their 28 patients (3 day *n* = 12; 5 day *n* = 8; placebo *n* = 8), there was a significant reduction in seizure frequency at one and two months post-cathodal tDCS vs. baseline in all three arms of the study. There was significant mean seizure frequency reduction in both three and five day cathodal tDCS as compared with placebo at two months follow-up (48% reduction in the treatment group vs. 6.3% reduction in the placebo group). There was significantly increased reduction in seizures in both the three days cathodal tDCS group (43% reduction in the treatment group vs. 6.3% reduction in the placebo group and five days group (55% reduction in the treatment group vs. 6.3% reduction in the placebo group). Short-term interictal epileptiform discharges were also significantly reduced after stimulation in all groups. There were limited side effects with this mode of treatment, mostly local sensory discomfort and mild headaches [72]. 

tDCS can be used to manage both focal and generalized epilepsy in both children and adults and provides a slightly cheaper (vs. repetitive transcranial magnetic stimulation (rTMS)), portable and alternative mode of treatment especially in the younger population who cannot tolerate rTMS. The studies for tDCS are limited and the majority of investigations are preliminary, however much promise is seen. 

### 3.3. Vagal Nerve Stimulation (VNS)

The first report of VNS was by Schwetzer and Wright in 1937, who looked at the effects of the knee jerk and various physiological changes in circulation and respiration by stimulating vagal afferents [73]. In 1997, the US FDA approved VNS as the first neuromodulation mode of treatment for drug-resistant epilepsy. In VNS surgery, the left vagus nerve, due to decreased cardiac side effects compared to the right, is stimulated. The stimulation of mostly (80%) afferent fibers is seen to converge onto the nucleus tractus solitarius, after which it proceeds onto the locus coeruleus [74]. Functional neuroimaging and electrophysiological studies of VNS have examined several areas of the brain (thalamus, cerebellum, orbitofrontal cortex, limbic system, hypothalamus, and medulla) and their levels of immediate response following stimulation. Albeit extensive, the clinical and animal VNS research remains inconclusive. It has been hypothesized that synaptic connections may be altered via VNS, modifying the electrical network in the brain [75]. Long-term VNS has been suggested to transform specific subcortical locations which in turn influence larger areas of the cortex [75,76,77].

Five RCTs investigating the efficacy of VNS were published between 1994 and 2014 [78,79,80,81,82]. The blinded phases of two key RCTs ultimately led to the approval of this mode of therapy by the FDA in epilepsy patients with drug-resistant epilepsy. These two RCTs showed that seizure frequency decreased >50% in 23–31% in the treatment groups vs. 13–15% in the control groups [78,79]. The study by Klinkenberg et al. is the only pediatric RCT to date [80]. This study demonstrated no significance in responder rate (>50% reduction in seizure frequency) with rates of 16% in high and 21% in low stimulation. One RCT compared VNS and best medical therapy vs. medical therapy alone, with no significant reduction in seizure frequency nor any difference in responder rates between treatment and control groups [81]. Aihua et al. explored the efficacy and safety of transcutaneous vagus nerve stimulation (tVNS) in patients with drug-resistant epilepsy. After 12 months, the monthly seizure frequency was lower in the tVNS group than in the control group (8.0 to 4.0; *p* = 0.003). All patients had improved Self-Rating Anxiety Scale, Self-Rating Depression Scale, Liverpool Seizure Severity Scale, and Quality of Life in Epilepsy Inventory-31 scores with minimal adverse effects including dizziness and drowsiness [82]. Other studies have stated complications of VNS placement included hoarseness (30%), dyspnea (13%), infection (12%), cough (7%), and throat pain (7%) [78,79,80,81,82]. 

### 3.4. Trigeminal Nerve Stimulation (TNS)

Following the treatment effects of VNS in epilepsy and evidence showing its mechanisms involving the locus ceruleus and nucleus solitarius, the trigeminal nerve was seen as a potential target since both the locus ceruleus and nucleus solitarius project onto the trigeminal nucleus [83,84,85]. External TNS (eTNS) is a noninvasive mode of treatment delivered by stimulating the trigeminal sensory roots within the facial tissue. Similarly to VNS, TNS is thought to produce an arousal-like effect via triggering the reticular activating system causing cortical and thalamic desynchronization [86].

DeGorgio et al. evaluated the safety and efficacy of eTNS in patients with drug-resistant epilepsy using a double-blind RCT design, to test the suitability of treatment and control parameters in preparation for a phase III multicenter clinical trial [87]. The responder rate, defined as >50% reduction in seizure frequency, was 30.2% for the eTNS group (120 Hz) vs. 21.1% for the active control group (2 Hz) for the 18-week treatment period (not significant, *p* = 0.31) [87]. The seizure frequency as measured by response ratio improved within each group compared to baseline. However, no differences were seen between the treatment and control groups. There was improvement in the patients’ depression within and between groups (Beck Depression Inventory score change of −8.13 in treatment group vs. −3.95 in the control group). Although eTNS may not seem so efficacious, there are still advantages to this treatment that one should consider. It is non-invasive and more economical. This RCT provides preliminary evidence that eTNS is safe and may be effective in subjects with drug-resistant epilepsy. Side effects are primarily limited to anxiety, headache, and skin irritation. Patients may also benefit from dual stimulation due to the central connections of the crossed and uncrossed pathways of the trigeminal nerve and the links to key subcortical structures such as the locus ceruleus, ascending reticular activating system, and projections to other subcortical nuclei [88]. 

### 3.5. Repetitive Transcranial Magnetic Stimulation (rTMS)

Developed in 1985 in the United Kingdom, TMS was used to examine cortical excitability in various epilepsy syndromes, the antiepileptic medication effects on the brain and to interrogate areas of the brain for potential surgery [89]. Once rTMS could be used to excite or suppress neural activity for prolonged periods of time, studies began to look at its potential use of TMS as a treatment modality for epilepsy. TMS is an electrode free-electrical stimulator, which uses alternating magnetic fields in order to create electrical currents that in turn stimulate regional neurons to improve epilepsy symptomatology. The magnetic pulse stimulates a small area of cortical tissue, depolarizing nearby axons [90]. Repetitive stimulation is seen to lengthen the effects of depolarization, maintaining its effect for more than an hour post-treatment [91]. Other than targeting the specific area of interest via anatomical positioning over the patient’s head, there is no way to target specific cell types, nor the interactions between inhibitory and excitatory cells. However, repetitive low-frequency stimulation has been hypothesized to cause prolonged inhibition as each pulse arrives during the late inhibitory phase of the last pulse, thus abating cortical hyper-excitability [90]. 

There has been a single double-blinded RCT of rTMS in patients with malformations of cortical development (MCD). Patients underwent five consecutive low-frequency (1 Hz, 1200 pulses) rTMS sessions targeting the MCD foci [92]. There was a significant reduction in seizure frequency in the rTMS group (−58% from baseline) vs. sham group (no change) as well as a significant reduction in epileptiform discharges straight after treatment (−31% from baseline) and at four weeks (−16% from baseline) vs. sham group (no change). There was improvement in subjective measures of social interaction and energy level and cognition in the treatment group vs. control. No serious adverse effects were reported. Headaches were experienced both in the treatment (25%) and control (22%) groups. There was no worsening of seizures. One patient in the control group reported insomnia [92]. Other open-label studies have shown reductions in seizure frequency with pathologies near the cortex in frontal and centro-parietal epilepsy, whereas targeting deeper structures in mesial temporal epilepsy has been associated with poor efficacy [93,94,95]. The majority of studies however have only shown evidence of decreasing epileptiform discharges, with no significant change in seizure reduction. A reason for such inconsistencies can be due to the selection bias, blinding bias, and differences in stimulation parameters [93,94,95].

rTMS is a non-invasive, inexpensive, pain-free procedure that has can modulate cortical brain activity. Evidence shows that rTMS is effective at abating epileptiform discharges, however the evidence for seizure reduction is still inconclusive. rTMS can certainly provide an alternative mode of treatment when considering foci lying over eloquent cortex that is not appropriate for surgery. However, further well-designed RCTs looking into the efficacy of this treatment are required to determine optimal stimulation frequencies, duration of treatment, intensity, and even the shapes of the magnet we use.

### 3.6. Responsive Neurostimulation (RNS)

The FDA approved RNS System (NeuroPace, Mountain View, CA, USA) is the first intracranial closed-loop system providing responsive stimulation directly to one or two seizure foci. Real time abnormal electrographic activity is detected and an automatic responsive stimulation is triggered thus halting any evolving seizure activity from propagating. Detection and stimulation parameters may be adjusted according to clinical benefit and to minimize side-effects related to stimulation. With the aforementioned possible mechanisms of stimulation in treating epilepsy, the RNS system is more amenable to the physiological transformations occurring during stimulation, thus potentially providing a better treatment modality over nonresponsive or continuous stimulation. 

In a large multi-centered double-blinded RCT, 191 patients who had drug-resistant focal seizures (defined in this RCT as failed ≥2 antiepileptic medication trials, ≥3 seizures/month, and 1 or 2 seizure foci) were implanted with an RNS system [96]. Morrell et al. reported 37.9% reduction in seizure frequency in the treatment arm vs. 17.3% reduction in the sham group at the end of the blinded phase [76]. However, no difference in responder rates was seen between the treatment and sham groups during the blinded phase of the study; additional reductions in seizure frequency in the treatment group to 44% at one year and 53% at two years were reported during the open-label extension [96,97]. Complications of RNS include a 4.7% rate of intracerebral hemorrhage and a 9% rate of infection after a mean of 5.4 years of follow-up, requiring neurostimulator explantation in 4.7% of the cases [96,97]. The six-year long-term analysis from this trial has shown that RNS mitigated substantial and sustained seizure reduction in their cohort of 111 patients with drug-resistant mesial temporal lobe epilepsy [98]. Using last observation carried forward (LOCF) analyses a median of 70% (interquartile range 31.8–92.9%; *n* = 106) seizure reduction was seen, and 50% responder rates of 66% (95% CI 56.6–74.4%) at six years. Forty-five percent (50/111) of patients reporting a seizure free period of ≥3 months, 29% (32/111) of patients ≥6 months, and 15% (17/111) of patients ≥ 1 year. It was also noted that the seizure reduction did not correlate with clinical characteristics such as mesial temporal sclerosis, bilateral seizure onset, and prior respective surgery/VNS operation. This treatment continues to improve with each year of implantation, and thus there is true potential for true neuromodulation and slow improvement [98]. In this same cohort, seizure reduction response in those with partial-onset seizures arising from eloquent cortex was investigated [99]. Over two-to-six year period post-implantation, a median seizure reduction of 70% was seen in frontal onset seizures, 58% in temporal neocortex and 51% in those of multilobar onset (LOCF analysis). It was also noted that therapeutic stimulation of eloquent cortex could be given sub-threshold and not exhibit side effects such as involuntary motor movement of altered motor performance when stimulating the primary motor cortex [99].

In addition, some recent open label studies with chronic continuous stimulation have also exhibited good outcomes [100,101,102]. Child et al. produced a proof of concept paper to show that continuous neocortical neurostimulation could provide significant reduction in seizure frequency, especially in those ineligible for resective surgery due to focal epilepsy arising from eloquent cortex [100]. Valentin et al. exhibited >90% seizure frequency reduction with one patient experiencing resolution of epilepsia partialis continua [101]. Lundstrom et al., in their cohort of 13 patients that underwent subthreshold cortical stimulation, showed suppression of interictal epileptiform discharges and improvement in clinical seizures [102].

RNS is a promising treatment for focal epilepsies even in bilateral disease and seizure foci affecting eloquent cortex. RNS, in comparison to the open-loop DBS and VNS, has a longer battery life due to a lower dose of stimulation. Stimulation is also not felt by patients, even when placed in an eloquent area, due to its low intensity. RNS is generally offered to those patients who are not suitable for resective surgery and who have foci in one or two areas of the brain. Further studies are necessary to optimize seizure detection, improve stimulation parameters, and build more contacts for cases of multiple foci.

## 4. Conclusions

Neuromodulation is a treatment strategy that is being used increasingly in those suffering from drug-resistant epilepsy that is not suitable for resective surgery. We are seeing more double-blinded RCTs demonstrating the efficacy of neurostimulation seizure patients. Although reductions in epilepsy frequency and focus firing are common in these trials, obtaining seizure freedom is rare. Invasive neuromodulation procedures (DBS, VNS, and RNS) have been approved as treatment measures. However, further investigations are necessary to delineate effective targeting, minimize side effects that are related to chronic implantation and to improve the cost effectiveness of these devices. The RCTs involved in the non-invasive modes of treatment whilst showing much promise (tDCS, eTNS, rTMS), have had only small recruitment numbers within their trials. Thus, they have not been sufficiently large enough to provide strong evidence on efficacy. Certainly, larger studies are needed, as well as studies that focus on better targeting techniques. 

## Figures and Tables

**Table 1:** Summary of double-blinded randomized controlled trials of neuromodulation in the management of drug resistant epilepsy.

Randomized Controlled Trials of VNS							
Study, Year	Country	Intervention	Study Setting	Population	Results	Follow Up	Complications
Ben-Menachem et al. 1994 [78]	Sweden	High vs. low stimulation treatment	Multicenter, Children and adults	- Drug resistant seizures - Numbers—High: *n* = 54 vs. Low: *n* = 60 - Mean age—High: 33.1 years vs. Low: 33.5 years - Females—High: 39% vs. Low: 37% - Mean duration—High: 23.1 years vs. Low: 20 years	- Reduction in seizure frequency—High: 24.5% vs. Low: 6.1% (*p* = 0.01) - At least 50% reduction in seizure frequency—High: 31% vs. Low: 13% (*p* = 0.02) - No seizure free patients - Seizure types were not significant - Implant was well tolerated	- 12 weeks post 2-week recovery period from surgical implantation	- Hoarseness (33%) - 1 patient died from myocardial infarction - 1 patient developed total vocal cord paralysis
Handforth et al. 1998 [79]	USA	High vs. low stimulation treatment	Multicenter, Children and adults	- Drug resistant focal impaired awareness seizures (patient had at least focal seizures over 30 days or focal seizures to bilateral tonic-clonic seizures) - Numbers—High: *n* = 95 vs. Low: *n* = 103 - Mean age—High: 32.1 years vs. Low: 34.2 years - Females—High: 48% vs. Low: 57% - Mean duration—High: 22.1 years vs. Low: 23.7 years	- Reduction in seizure frequency—High: 27.9% vs. Low: 15.2% (*p* = 0.04) - No difference in between-group comparison for 50% responders—High: 23.4% vs. Low: 15.7% - One patient seizure free (High) - No change in physiologic indicators of cardiac or pulmonary function	- 12–16 weeks after 2-week ramp-up period	- Hoarseness (30%) - Dyspnea (13%) - Infection (12%)
Klinkenberg et al. 2012 [80]	Netherlands	High vs. low stimulation treatment for 20 weeks, then all received high for 19 weeks	Single center, Children	- 41 children total (35 with focal epilepsy: 25 structural, 10 unknown etiology; 6 with generalized epilepsy) - Numbers—High: *n* = 21 vs. Low: *n* = 20 - Mean age—High: 10 years 11 months vs. Low: 11 years 6 months - Mean duration—High: 7 years 8 months vs. Low: 9 years 5 months - Seizure frequency and severity were recorded using diaries and the adapted Chalfont Seizure Severity Scale	- Reduction in seizure frequency at least 50%—High: 16% vs. Low: 21%	- 20 weeks	- Voice alteration (20%) - Coughing (7%) - Throat pain (7%) - Infection (5%)
Ryvlin et al. 2014 [81]	France	PuLsE (Open Prospective Randomized Long-Term Effectiveness): VNS + Best medical practice (BMP) vs. BMP	Multicenter, Adults–early termination of trial due to low enrollment	- Drug resistant focal seizures (available baseline data and ≥1 post-op QOLIE-89: 48 with VNS + BMP, and 48 with BMP alone) - Mean age—Treatment group: 38 years vs. Control group: 41 years - Females—Treatment group: 50% vs. Control group: 44% - Mean duration—Treatment group: 25 years vs. Control group: 25 years	- Significant improvements in HRQoL (QOLIE-89)—Treatment group (VNS+BMP): 5.5 points vs. Control group (BMP): 1.2 (*p* < 0.05) - No difference in secondary endpoints: Seizure frequency Responder rate CES-D NDDI-e AEP - AED Load	- 24 months in seven patients, 12 months in 60.	- Transient vocal cord paralysis (4%) - Brief period of respiratory arrest (3%)
Aihua et al. 2014 [82]	China	Transcutaneous: Ramsay Hunt zone stimulation (treatment group) vs. earlobe (control stimulation)	Single center, Children and adults	- Numbers—60 patients randomly divided into two groups based on stimulation zone - Mean age—Treatment group: 34.5 years vs. Control group: 29.0 years - Mean duration—Treatment group: 10.7 years vs. Control group: 17.6 years - Seizures types—Treatment group: focal aware (65%), focal impaired awareness (11%), generalized (23%) vs. Control group: focal onset aware (71%), focal impaired awareness (14%), generalised (14%)	- Reduction in seizure frequency at 12 months—Treatment group: 8/month vs. Control group: 4/month (*p* = 0.003) - Antiepileptic drugs were maintained at a constant level in all subjects. - All patients showed improved SAS, SDS, LSSS, QOLIE-31 scores	- 12 months	- Dizziness (3%) - Drowsiness (9%)
**Randomized controlled trials of DBS**							
Van Buren et al. 1978 [21]	USA	Bilateral stimulation of the superior surface of the cerebellum. Treatment group: 10–14 V, 10 Hz vs. off stimulation	Single center, Adults	- Drug-resistant epilepsy - Numbers—5 - Mean age—27.2 years (18–34) - Mean duration—8 to 23 years	- No significant differences in seizure frequency were identified	- Up to 1 or more weeks (total 52 days) of blinded phase over 15–21 months	- No complications mentioned
Wright et al. 1984 [22]	United Kingdom	Stimulation of the upper surface of the cerebellum 2 cm from midline on each side Treatment group: 1–7 mA, 10 Hz in either continuous or contingent session vs. sham stimulation	Single center, Adults	- Drug-resistant epilepsy - Numbers—12 - Mean age—30 years (20–38) - Female—17% - Mean duration—10 to 32 years	- No reduction in seizure frequency occurred that could be attributed to stimulation - Cerebellar stimulation is not recommended	- 6-month blinded phase consisted of 3 × 2 month periods	- Infection with electrodes removal (16.7) - Electrode displacement required reoperation (25%) - Lead pain that required repositioning (8.3%) - Apparatus failure (8.3%) - Receiver pocket burst (8.3%)
Velasco et al. 2005 [23]	Mexico	Cerebellar stimulation -bilateral modified four-contact plate electrodes adjusted to 2.0 μC/cm^2^/phase	Single center, Adults	- Drug-resistant focal motor seizures - Numbers—*n* = 5 (*n* = 3 with stimulation ON and *n* = 2 with stimulation OFF in blinded phase) - Randomized blinded phases for 3 months followed by all ON stimulation - Patients served as own controls (Compared seizure frequency pre-implant (3 months) vs. post-implant phases (average, eight epochs of 3 months each)	- Reduction in seizure frequency—Stimulation ON: GTCs to 33% vs. OFF: no change (at 3 months) (patient 2, 21%; patient 3, 46%; patient 4, 32%) (*p* = 0.023) Open label for 6 months: - Mean seizure rate of 41% of the baseline	- 3 months	- 1 infection that required implant removal
Fisher et al. 1992 [26]	USA	Bilateral stimulation of the centromedian thalamic nucleus (0.5 to 10 V, 65 Hz, 90 µs pulse width) vs. sham stimulation	Multicenter, Adults	- Drug-resistant focal epilepsy (1), focal epilepsy with generalization (1), generalized epilepsy (5) - Numbers—7 - Mean age—28 - Female—57.1% - Mean duration—14 to 29 years	- Reduction in seizure frequency—treatment group: 30% reduction vs. 8% sham stimulation - During open label time, 3 patients reported 50% decrease in seizure frequency	- 3 months period of either treatment or sham stimulation with a 3-month washout phase between - Followed by 3–13 months open label with all on	- No complications mentioned
Velasco et al. 2000 [27]	Mexico	Alternate stimulation between the left and the right centromedian thalamic nucleus (4–6 V, 60 Hz, 450 µs pulse width) vs. sham stimulation	Single center, Adults and children	- Lennox-Gastaut syndrome with atypical absences and GTCS (8), complex partial and secondary generalized (5) - Numbers—13 - Mean age—19.2 years - Female—38% - Mean duration—4 to 33 years	- Total number of seizure, GTCS, absence and CPS were reduced significantly (absolute and relative percentage decrease) turning off stimulation did not cause a return to base line levels - No statistical significant differences between the on and off periods (*p* = 0.23) - 5 out of 11 patients with GTCS and 2 out of 8 patients with absence seizures were seizure free	- 6–9 months period of stimulation followed by a 6-month cross-over pairs: 2 × 3 month phase On/Off or Off/On - Followed by 42 months follow up	- 1 death due to herpes encephalitis
Fisher et al. 2010 [6]	USA	Anterior nuclei of thalamus stimulation	Multicenter, Adults	- Drug resistant focal seizures - Numbers—Treatment group = 54 vs. Control group = 5 - Mean age—Treatment group: 35.2 years vs. Control group: 36.8 years - Female—Treatment group: 54% vs. Control group: 46% - Mean duration—Treatment group: 21.6 years vs. Control group: 22.9 years	- Reduction in seizure frequency—Treatment group: 29% greater reduction vs. control group (last month of blinded trial) - Responder rate 54% by 2 years - 14 patients seizure free for at least 6 months	- 3 months blinded followed by 9 months open label with all on - 5-year follow-up study (Salanova)	- Paresthesias (22.7%) - Implant site pain (20.9%) - Implant site infection (12.7%)
Tellez– Zenteno et al. 2006 [52]	Canada	Left hippocampal stimulation (1.8 V to 4.5 V, 190 Hz, 90 µs pulse width) vs. sham stimulation	Single center, Adults	- Drug-resistant left unilateral MTLE - Numbers—4 - Mean age—31.8 years - Female—75% - Mean duration—16 to 24 years	- A median reduction in seizure frequency during treatment—15% - Seizure improved in three patients, but the result was not statistically significant	- 3 × 2-month treatment pairs with monthly phase On or Off	- No complications mentioned
Velasco 2007 et al. [53]	Mexico	Bilateral or unilateral hippocampal stimulation Treatment group: 130 Hz, 450 μs pulse width vs. control group: No stimulation	Single center, Children and adults	- Drug-resistant CPS and GTCS - Numbers—Treatment group: *n* = 4 vs. Control group: *n* = 5 - Age—Treatment group: 20–40 years vs. Control group: 14–43 years - Female—Treatment group: 25% vs. Control group: 40% - Mean duration—Treatment group: 12.0 years vs. Control group: 10.4 years	- Seizure reduction in treatment group vs. baseline seizure frequency in control group, proving that the initial seizure decrease is not due to electrode implantation effect. - 18 months follow up: >95% seizure reduction in 5 patients with normal MRI, and 50–70% seizure reduction in 4 patients with hippocampal sclerosis	- 1-month blinded phase followed by 18–84 months open label with all on	- Skin erosion with local infection in 3 patients, one of which required plastic surgery and eventual electrode removal
McLachlan et al. 2010 [54]	Canada	Bilateral hippocampal stimulation Treatment group: 185 Hz, 90 µs pulse width	Single center, Adults	- Drug-resistant focal epilepsy with bitemporal origination - Numbers—2 - Age—45 and 54 years - Female—50% - Mean duration—15 and 29 years	- Reduction in seizure frequency by 33% in the two patients during stimulation - Reduction in seizure frequency by 25% for the 3 months post stimulation before return to baseline (*p* < 0.01)	- 3 months period of stimulation On/Off followed by 3 months washout and repeat cycle	- No complications noted
Wiebe et al. 2013 [55]	Canada	Hippocampal stimulation, unilateral or bilateral (Treatment group: 135 Hz continuous cathodal stimulation of all electrodes involved in seizure generation vs. control group: no stimulation)	Multicenter, Adult	- Drug-resistant MTLE - Numbers—Treatment group: 2 patients vs. Control group: 4 patients - Mean age—Treatment group: 30 years vs. Control group: 35–46 - Baseline seizure frequency—Treatment group: 12 seizures per month vs. Control group: 10 seizures per month	- Statistically nothing significant - Mean seizure reduction: Treatment group: 45% vs. Control group: 60% increase - Half of the patients in treatment group had >50% reduction. - Improvement with hippocampus sclerosis in the frequency of all types of seizures, and in subjective memory function - Borderline significant improvement in attention/concentration - Recall function worse	- 7 months	- No complications mentioned
Cukiert et al. 2017 [56]	Brazil	Hippocampal stimulation, unilateral or bilateral (Treatment group: active stimulation at continuous 130 Hz, duration 300 μs, final intensity of 2 V (0.4 V increments) vs. control group: no stimulation)	Single center, Children and adults	- Drug resistant temporal lobe epilepsy - Numbers—Treatment group: 8 patients vs. Control group: 8 patients - Mean age—38.4 years - Mean pre-operative seizure frequency 12.5/month	- Treatment group: 50% of patients seizure free, 87.5% had >50% seizure reduction - Significant reduction in seizures (focal impaired awareness) from first month to end of blinded phase - Significant reduction in seizures (focal awere) except 3rd month of blinded phase	- 6 months blinded phase	- Local skin erosion (12.5%)
Kowski et al. 2015 [63]	Germany	Bilateral stimulation of nucleus accumbens and the anterior thalamic nuclei Treatment group: 5 V, 125 Hz, 90 µs pulse width	Single center, Adults	- Inclusion criteria: 3 major seizures every 4 weeks during 3 month period - Numbers—4 - Mean age—36.7 - Female—75% - Mean duration—12.5 years	- Reduction in seizure frequency >50% in 3 patients	- 3 months period of stimulation On/Off, followed by 1 month washout and repeat cycle - 3-month open label - Anterior thalamic nuclei always stimulated	- 1 infection that required implant removal, but a patient re-participated in the study after clearance of infection
San-Juan et al. 2017 [72]	Mexico	Transcranial direct current stimulation (tDCS)–(randomized into three treatment arms: 2 mA cathodal direct current stimulation for 30 min: (1) three days (2) five days vs. (3) placebo)	Multicenter, Adults	- Drug-resistant MTLE with hippocampal sclerosis - Numbers—*n* = 28 - Mean age—37.8 years	- Reduction in seizure frequency—Treatment groups: 48% vs. Placebo group: 6.3% (at 2 months) (*p* = 0.008)	- 2 months	- 2 patients had a focal impaired awareness seizure towards the end of first day session, however not believed to be intervention-related given high baseline seizure frequency
Velasco et al. 2005 [23]	Mexico	Cerebellar stimulation-bilateral modified four-contact plate electrodes adjusted to 2.0 μC/cm^2^/phase	Single center, Adults	- Drug-resistant focal motor seizures - Numbers—*n* = 5 (*n* = 3 with stimulation ON and *n* = 2 with stimulation OFF in blinded phase) - Randomized blinded phases for 3 months followed by all ON stimulation - Patients served as own controls (Compared seizure frequency pre-implant (3 months) vs. post-implant phases (average, 8 epochs of 3 months each)	- Reduction in seizure frequency—Stimulation ON: GTCs to 33% vs. OFF: no change (at 3 months) (patient 2, 21%; patient 3, 46%; patient 4, 32%) (*p* = 0.023) Open label for 6 months: - Mean seizure rate of 41% of the baseline	- 3 months	- 1 infection that required implant removal
**Randomized controlled trial of TNS**							
DeGiorgio et al. 2013 [87]	USA	External trigeminal nerve stimulation (eTNS)–treatment group: eTNS 120 Hz vs. control group: eTNS 2 Hz	Multicenter, Adults	- At least 2 focal seizures/month - Numbers—Treatment group: *n* = 25 vs. Control group: *n* = 25 - Mean age—Treatment group: 33.1 years vs. Control group: 34 years - Female—Treatment group: 64% vs. Control group: 44% - Mean duration—Treatment group: 16.7 years vs. Control group: 12.0 years	- No difference in responder rate—Treatment group: 31% vs. Control group: 21.1% (*p* > 0.05) - Improved seizure frequency within each group as measured by response ratio, but no difference between treatment vs. control group - Improvement in depression—Treatment group: BDI score change of −8.13 vs. Control group: BDI score change of −3.95 (*p* = 0.002)	- 18 weeks	- Skin irritation (14%) - Anxiety (4%) - Headache (4%)
**Randomized controlled trial of rTMS**							
Fregni et al. 2006 [92]	USA	Repetitive transcranial magnetic stimulation (rTMS)–treatment group: 1 Hz, 1200 pulses vs. sham group	Single center, Adults	- MCD - Numbers—Treatment group: *n* = 12 vs. Sham group: *n* = 9 - Mean age—Treatment group: 21.3 vs. Sham group: 22.7	- Reduction in seizure frequency—Treatment group: 58% reduction vs. Sham group: No difference from baseline - In treatment group only: significant decrease in the number of epileptiform discharges immediately post treatment (*p* = 0.01) and at week 4 (*p* = 0.03)	- 60 days	- Headache (Treatment group: 5% vs. Sham group: 22%) - Insomnia (11%)
**Randomized controlled trial of RNS**							
Morrell et al. 2011 [96]	USA	Responsive neuromodulation	Multicenter, Adults	- Failed ≥2 antiepileptic medication trials, ≥3 seizures/month, and 1 or 2 seizure foci - Numbers—Active stimulation: *n* = 97 vs. Sham stimulation *n* = 94 - Mean age—Active stimulation: 34.0 vs. Sham stimulation: 35.9 - Female—Active stimulation 48% vs. Sham stimulation 47% - Mean duration—Active stimulation: 20.0 years vs. Sham stimulation 21.0 years	- Reduction in seizure frequency—Active stimulation: −37.9% vs. Sham stimulation: −17.3% (*p* = 0.012) during blinded period - Responder rate—Active stimulation: 29% vs. Sham stimulation: 27% - 2 cases in active stimulation group were seizure free for the blinded phase - QOLIE-89 scores improved in active and sham stimulation, continued through 1 and 2 years Open label: - Median % reduction in seizure frequency of 44% (1st year), 53% (2nd year) - Statistically significant improvement in QOLIE scales at 1 and 2 years (*p* < 0.05)	- 12-week blinded period followed by 84-week open-label period	- Intracerebral hemorrhage (4.7%) - Infection (5.2% at end of open-label phase, 9.0% after mean follow-up 5.4 years of which 4.7% underwent explantation)

VNS (Vagus Nerve Stimulation); DBS (Deep Brain Stimulation); TNS (Trigeminal Nerve Stimulation); rTMS (Repetitive Transcranial Magnetic Stimulation); RNS (Responsive Neurostimulation); CES-D (Center for Epidemiologic Studies Depression Scale); NDDI-e (Neurological Disorders Depression Inventory–Epilepsy); AEP (Adverse Event Profile); AED (Antiepileptic Drug); SAS (Self-Rating Anxiety Scale); SDS (Self-Rating Depression Scale); LSSS (Liverpool Seizure Severity Scale); MTLE (Mesial Temporal Lobe Epilepsy); MCD (Malformation of Cortical Development); QOLIE-31 (Quality of Life in Epilepsy Inventory); BMP (Best Medical Practice); HRQoL (Health-Related Quality Of Life).
